# Critical Errors in Inhaler Technique Among Patients With Asthma and COPD: A Cross‐Sectional Study in a Real‐World Setting

**DOI:** 10.1155/carj/8222875

**Published:** 2026-09-26

**Authors:** Italo Henrique Medeiros Damasceno, Ricardo José Fonseca De Oliveira, Sabrina Joany Felizardo Neves, Rand Randall Martins, Rodrigo Dos Santos Diniz

**Affiliations:** ^1^ Postgraduate Program in Pharmaceutical Services and Policies, Health Science Center, Federal University of Rio Grande Do Norte, Natal, Brazil, ufrn.br; ^2^ University Hospital Onofre Lopes, Health Science Center, Federal University of Rio Grande Do Norte, Natal, Brazil, ufrn.br; ^3^ Institute of Pharmaceutical Sciences, Federal University of Alagoas, Maceió, Brazil, ufal.edu.br; ^4^ Faculty of Pharmacy, Health Science Center, Federal University of Rio Grande Do Norte, Natal, Brazil, ufrn.br

**Keywords:** asthma, COPD, critical errors, inhalation devices, inhaler technique

## Abstract

**Background:**

The correct use of inhalation devices is essential for achieving adequate control of asthma and COPD; however, critical errors in inhalation technique are frequent and substantially reduce treatment effectiveness. This study’s objective was to evaluate the prevalence of inadequate inhalation technique among patients with asthma and COPD and to identify the most common critical errors and the factors associated with incorrect use.

**Methods:**

This cross‐sectional observational study included 575 patients (aged ≥ 18 years) with asthma and/or COPD, recruited from a university hospital outpatient pulmonology clinic and a public specialized pharmacy (Natal, Brazil). Participants completed sociodemographic questionnaires and demonstrated their inhaler technique, which was assessed using device‐specific checklists to identify critical errors. Factors associated with critical errors were analyzed using multivariable logistic regression.

**Results:**

Only 8.2% of participants correctly performed all steps of the inhalation technique. At least one critical error was observed in 73.4% of the cohort. In multivariable analysis, advanced age (OR: 1.029; 95% CI: 1.016–1.044), use of multiple devices (OR: 2.500; 95% CI: 1.754–3.562), and type of mechanism independent of inspiration (OR: 0.262; 95% CI: 0.160–0.430; protective factor) were significantly associated with critical errors (*p* < 0.001).

**Conclusions:**

Inadequate inhalation technique, particularly the occurrence of critical errors, was highly prevalent, especially among older patients and those using multiple devices. Inhalers dependent on inspiratory effort demonstrated greater vulnerability to incorrect use.

## 1. Introduction

Asthma and chronic obstructive pulmonary disease (COPD) are highly prevalent chronic respiratory conditions worldwide, associated with substantial morbidity and mortality [1]. Regarding available therapeutic options, inhaled medications are considered the cornerstone of treatment, as they enable direct delivery of the drug to the lungs, thereby maximizing therapeutic effects while minimizing potential adverse events related to systemic administration. However, the effectiveness of this approach critically depends on the correct inhaler technique by patients [2].

Incorrect use of inhaler devices is a highly prevalent problem among patients with chronic respiratory diseases, particularly those with asthma and COPD [3, 4]. Errors in inhaler technique result in suboptimal drug deposition in the airways, thereby compromising symptom control and increasing the risk of exacerbations, hospitalizations, and healthcare costs [5]. The identification of inhaler technique errors is a critical first step in optimizing treatment and can be performed using assessment tools tailored to the specific type of device. However, the literature reports considerable variability in the prevalence of these errors, ranging from 50% to 100%. This heterogeneity is partly explained by the wide variety of inhaler devices and the use of different assessment tools across studies [6].

A more systematic approach to characterizing inhaler technique errors is to focus on critical errors, defined as failures in essential steps of the inhalation process that may negate treatment effectiveness, such as exhalation prior to inhalation, adequate sealing around the mouthpiece, proper inhalation, and postinhalation breath‐hold [7, 8]. Although several studies have described the prevalence of incorrect inhaler use, many have primarily focused on overall technique errors, without specifically emphasizing clinically relevant critical errors across different inhaler devices. Furthermore, there is limited evidence regarding patient‐ and device‐related factors associated with the occurrence of these errors, particularly in real‐world settings in Latin American countries, where studies addressing this issue remain scarce [9, 10]. Therefore, the aim of this study is to assess the adequacy of inhaler device use in patients with asthma and COPD, with a focus on identifying critical inhaler technique errors and the factors associated with their occurrence.

## 2. Methods

### 2.1. Study Design and Population

This was a cross‐sectional observational study conducted between May 2022 and July 2024, including patients with asthma or COPD using inhaler devices. Participants were recruited from two public healthcare services affiliated with the Brazilian Unified Health System (SUS), both located in Natal, Rio Grande do Norte, Brazil: (i) a specialized pulmonology outpatient clinic at a university hospital; and (ii) a public pharmacy specialized in the free dispensing of high‐cost medications. These sites were selected because they represent complementary points of care for patients receiving inhaled therapy. The university hospital outpatient clinic provides specialized pulmonology care, whereas the public pharmacy serves the metropolitan region of Natal and dispenses medications based on prescriptions originating from different levels of care, including both public and private healthcare services. Recruitment from both settings was intended to include patients with different pathways of access to respiratory care. A consecutive nonprobability sampling strategy was used, with eligible patients attending each recruitment site during the study period being consecutively approached for participation. Each participant was included only once in the study.

Individuals of both sexes, aged over 18 years, with a diagnosis of asthma and/or COPD, and prescribed one or more inhaler devices for maintenance therapy were eligible for inclusion. Patients with evident cognitive impairment (psychiatric or neurological) that could compromise data collection, as well as those requiring assistance with using inhaler devices, were excluded. The study was approved by the Institutional Review Board of the Januário Cicco Maternity School (protocol number 5.867.912/2022) and was conducted in accordance with the Declaration of Helsinki and the Brazilian National Health Council Resolution No. 466/2012. Written informed consent was obtained from all participants.

Based on an expected prevalence of inadequate inhaler technique of 32% [3], as reported in a previous systematic review and used as an approximate estimate of the occurrence of critical inhaler technique errors, the required sample size was calculated as 523 participants, considering a margin of error of ±4 percentage points and a 95% confidence level.

### 2.2. Data Collection

Participants were consecutively recruited and invited to take part in the study after providing written informed consent. Interviews were conducted in a private consultation room while participants were awaiting medical care. Respondents were asked about sociodemographic characteristics, current disease status, medication use, and inhaler technique.

Sociodemographic data included sex, age, household income, educational level, and occupation. Participants were also asked about smoking status and alcohol consumption. Clinical diagnosis of the respiratory condition and prescribed medications were obtained from electronic medical records. In addition, participants were questioned regarding unplanned healthcare utilization within the previous 3 months, defined as emergency department visits, hospital admissions, or urgent outpatient consultations due to respiratory exacerbations. Medication adherence was assessed using the Morisky–Green–Levine questionnaire [11], with participants classified as nonadherent if they provided at least one affirmative response to the four items.

Inhaler devices were classified according to their drug delivery characteristics as dry powder inhalers (DPIs; Aerocaps, Ellipta, and Diskus), pressurized metered‐dose inhalers (pMDIs), and soft mist inhalers (SMIs; Respimat). For analyses based on dependence on inspiratory effort, DPIs were categorized as breath‐actuated (inspiration‐dependent) devices, whereas pMDIs and SMIs were categorized as inspiration‐independent devices. DPIs require patients to perform a rapid and deep inhalation to generate sufficient inspiratory flow for adequate drug dispersion and delivery. In contrast, pMDIs use a pressurized propellant, and SMIs use a mechanical spring mechanism for drug release, independently of the patient’s inspiratory effort.

Participants were asked to identify the inhaler device they used most frequently and were invited to demonstrate their inhaler technique for assessment. This pragmatic approach was intended to reflect the device with the greatest daily exposure and presumed clinical relevance, simulating routine clinical reassessment during outpatient visits. Two previously trained assessors (IHMD and RSD) independently observed and evaluated the inhaler technique. For each device, a specific checklist was applied, as described in the Supporting Material, assessing the following steps: device preparation, exhalation, correct positioning of the device at the lips, inhalation, breath‐hold, exhalation, completion of the procedure, and device cleaning. These checklists were developed based on national and international recommendations for inhaler use, as well as previously published literature on inhaler technique assessment [12–15]. Assessors evaluated each step performed by the participant by marking “yes” or “no” for correct execution; each correct step was assigned one point, and a total score was calculated. All participants had their inhaler technique assessed for only one device, defined as the one most frequently used. In cases of disagreement between assessors, a third evaluator was consulted.

Among the identified execution errors, those with the greatest potential to compromise treatment effectiveness were classified as critical errors [6, 7]. These critical errors were identified from the same device‐specific checklists used for inhaler technique assessment, as they corresponded to key steps within the evaluation instrument that are considered essential for adequate drug delivery according to the literature. The critical errors evaluated in this study are described as follows:

1. Insufficient exhalation: failure to fully exhale before initiating inhalation through the device, which reduces the inspiratory volume and, consequently, the amount of medication reaching the lungs.
2. Incorrect mouthpiece positioning and seal: improper insertion of the inhaler mouthpiece into the oral cavity, including the absence of a firm lip seal, misalignment of the mouthpiece, or incorrect head posture during administration.
3. Insufficient inhalation: inhalation technique that is inappropriate in terms of speed, depth, or coordination with device actuation. This includes shallow, slow (when fast is required), or fast (when slow is required) inhalations, as well as failure to coordinate breathing with the actuation of the inhaler.
4. Absent or insufficient inspiratory breath‐hold: failure to perform a voluntary breath‐hold after inhalation or holding for less than 5 s (or less than the minimum duration recommended for the specific inhaler type).

Data were collected by the principal investigator (IHMD), with support from a team of undergraduate pharmacy students who had received formal training from a pulmonologist (RJFO) in standardized procedures for inhaler technique assessment and study data collection. These students assisted with participant recruitment and the administration of the sociodemographic questionnaire. Prior to data collection, a pilot study involving 10 individuals was conducted to calibrate the study instruments and train the research team.

### 2.3. Statistical Analysis

Statistical analyses were performed using Stata software, Version 15.0 (StataCorp, College Station, TX, USA). Continuous variables were described as means and standard deviations, and categorical variables as absolute and relative frequencies. Overall comparisons of completely correct technique and the occurrence of at least one critical error across the evaluated inhaler devices were performed using Fisher’s exact test. A *p* value < 0.05 was considered statistically significant.

Inhaler technique performance was expressed as the percentage of checklist steps correctly completed relative to the total number of device‐specific steps, with corresponding 95% confidence intervals. The prevalence of participants presenting with one or more critical errors during technique demonstration was also estimated. Factors associated with the occurrence of critical errors were identified using univariable logistic regression, with calculation of odds ratios (ORs) and their respective 95% confidence intervals. Variables with *p* < 0.10 were included in the multivariable model, and final predictors were selected using a backward stepwise approach (*p* < 0.05). Multicollinearity was assessed using the variance inflation factor (VIF), with all values below 5, indicating the absence of relevant collinearity. To compare the prevalence of critical errors between devices classified according to drug delivery mechanism (breath‐actuated inhalers vs. inspiration‐independent inhalers), Pearson’s chi‐square test was applied.

## 3. Results

During the study period, 581 patients were invited to participate; 63 did not have any inhaler device available at the time of the interview, and six were deemed ineligible (one with a mental disorder and five dependent on family members for inhaler use). The final analyzed sample comprised 512 patients, including 156 from the pulmonology outpatient clinic and 356 from the public pharmacy. Sociodemographic and clinical characteristics are presented in Table 1. The mean age was 59.4 years, with 299 participants (58.4%) aged ≥ 60 years. Most participants were female (322, 62.9%), and a low educational level predominated, with 66.6% (341) having only primary education.

**TABLE 1:** Sociodemographic and clinical characteristics of the study population.

Parameters	Total population (*n* = 512)	
Age in years (*m*, sd)	59.4	15.1
Older adults (≥ 60 years)	299	58.4
Female sex (*n*, %)	322	62.9
Maximum monthly income up to one minimum wage (*n*, %)	100	19.5
Number of household members (*m*, sd)	2.8	1.5
Maximum education level: elementary school (*n*, %)	341	66.6
Retired or receiving pension (*n*, %)	287	56.1
Current smoker or passive smoker (*n*, %)	84	16.1
Former smoker (*n*, %)	251	49.0
Alcohol consumption (*n*, %)	115	22.5
Clinical diagnosis of respiratory disease (*n*, %)		
Asthma	357	69.7
Chronic obstructive pulmonary disease (COPD)	100	19.5
Asthma and COPD	12	2.3

*Note:* mean and standard deviation (*m*, sd); absolute and relative frequencies (*n*, %); minimum wage equivalent to R$1412.00 (MW); chronic obstructive pulmonary disease (COPD).

Table 2 presents the recent clinical history and inhaler use profile. Overall, 33.4% of patients reported seeking healthcare due to exacerbations within the previous 3 months, and 65.2% were classified as nonadherent to treatment according to the Morisky–Green–Levine test. The mean number of devices used per patient was 2.1 ± 0.7; most participants used two inhaler devices (326, 63.7%), while 84 (16.4%) used one device and 97 (18.9%) used three or more devices. DPIs were the most frequently used device category, predominantly Aerocaps (90.2%), followed by pMDIs (26.4%) and SMIs (Respimat, 9.0%). Most patients used DPIs, categorized as breath‐actuated devices (93.0%), whereas 33.2% used at least one inspiration‐independent device (pMDI or SMI). Although four participants reported using Diskus as part of their treatment regimen, none selected it as the device most frequently used; therefore, Diskus was not represented in the inhaler technique assessments.

**TABLE 2:** Recent clinical history, medication adherence, and inhaler device use profile.

Parameters	Values	
Health care utilization due to exacerbation in the past 3 months (*n*, %)	171	33.4
Nonadherence to medication (Morisky–Green–Levine test) (*n*, %)	333	65.2
Number of inhaler devices in use (*m*, sd)	2.1	0.7
Type of inhaler device (*n*, %)		
Aerocaps (dry powder inhaler—DPI)	462	90.2
Pressurized metered‐dose inhalers (pMDI)	135	26.4
Respimat (soft mist inhaler—SMI)	46	9.0
Diskus (dry powder inhaler—DPI)	4	0.8
Ellipta (dry powder inhaler—DPI)	23	4.5
Type of drug delivery mechanism (*n*, %)		
Breath‐actuated inhalers^∗^	476	93.0
Inspiration‐independent inhalers^∗∗^	170	33.2

*Note:* absolute and relative frequency (*n*, %); mean and standard deviation (*m*, sd).

^∗^DPI.

^∗∗^pMDI + SMI.

Table 3 details patient performance in executing inhalation technique according to the type of device used. In 3.5% of assessments, a third evaluator was consulted to reach consensus. The rate of completely correct technique, defined as 100% of steps performed correctly, was observed in only 8.2% of the sample. The overall mean performance was 69.6% (95% CI: 68.1–71.2), corresponding to the proportion of checklist steps correctly completed relative to the total number of required steps for each device. Among the evaluated devices, the lowest performance was observed among users of the DPI Ellipta users (mean accuracy: 54.4%; 0% with correct technique) and Respimat users (58.1%; 0% with correct technique), with all patients in these groups committing at least one critical error. The DPI Aerocaps, despite being the most commonly used device, showed a high rate of critical errors (76.9%), with only 6.6% of users performing all steps correctly. In contrast, pMDIs demonstrated the best overall performance, with a mean accuracy of 83.1% (95% CI: 79.7–86.5) and 23.0% of users achieving correct technique. Nevertheless, more than one‐third of pMDI users committed critical errors (37.7%). The overall prevalence of critical errors was 73.4%. The frequency of completely correct technique differed significantly across the evaluated inhaler devices (Fisher’s exact test, *p* = 0.001), as did the occurrence of at least one critical error (*p* < 0.001).

**TABLE 3:** Inhaler technique performance and occurrence of critical errors by device type.

Type of inhaler device	Evaluations		Correct technique (100% of steps completed correctly)		Correct steps (%)			One or more critical errors	
	*n*	%	*n*	%	Mean	95% CI		*n*	%
Breath‐actuated inhalers^∗^									
Aerocaps	424	83.0	28	6.6	68.7	66.9	70.4	326	76.9
Ellipta	13	2.5	0	0.0	54.4	45.7	63.2	13	100.0
Inspiration‐independent inhalers^∗∗^									
Pressurized metered‐dose inhalers	61	11.9	14	23.0	83.1	79.7	86.5	23	37.7
Respimat	14	2.7	0	0.0	58.1	52.5	63.9	14	100.0
Total	512	100.0	42	8.2	69.6	68.1	71.2	375	73.4

*Note:* absolute and relative frequencies (*n*, %); 95% confidence interval (95% CI). Overall comparisons across inhaler devices using Fisher’s exact test: completely correct technique, *p* = 0.001; occurrence of one or more critical errors, *p* < 0.001. Table 3 includes only the inhaler device selected for technique assessment, defined as the device most frequently used by each participant.

^∗^DPI.

^∗∗^pMDI + SMI.

Table 4 presents the results of the multivariable logistic regression analysis identifying factors associated with the occurrence of at least one critical error. Older age (OR: 1.029; 95% CI: 1.016–1.044; *p* < 0.001) and the use of a greater number of inhaler devices of different types (OR: 2.500; 95% CI: 1.754–3.562; *p* < 0.001) were independent risk factors. Conversely, the use of inspiration‐independent devices was a protective factor (OR: 0.262; 95% CI: 0.160–0.430; *p* < 0.001). Variables such as household size, educational level, employment status, former smoking, and asthma diagnosis were not significantly associated in the final model.

**TABLE 4:** Factors associated with critical inhaler technique errors: multivariable logistic regression model.

Parameters	Univariable analysis				Multivariable analysis			
	Odds ratio	95% CI		*p*	Odds ratio	95% CI		*p*
Age (years)	1.033	1.019	1.046	< 0.001	1.029	1.016	1.044	< 0.001
Female sex	0.935	0.622	1.405	0.745				
Maximum monthly income up to one minimum wage	1.127	0.658	1.929	0.663				
Number of household members	0.848	0.743	0.968	0.014				
Maximum education level: elementary school	1.724	1.149	2.585	0.008				
Retired or receiving a pension	1.888	1.269	2.808	0.002				
Current smoker or passive smoker	0.774	0.464	1.290	0.326				
Former smoker	1.662	1.115	2.478	0.012				
Alcohol consumption	1.326	0.802	2.195	0.272				
Asthma	1.811	1.198	2.736	0.005				
COPD	0.629	0.392	1.007	0.054				
Health care utilization due to exacerbation in the past 3 months	0.819	0.544	1.235	0.341				
Nonadherence to medication	0.980	0.649	1.480	0.922				
Number of inhaler devices in use	1.566	1.152	2.130	0.004	2.500	1.754	3.562	< 0.001
Breath‐actuated inhalers	3.025	1.524	6.005	0.002				
Inspiration‐independent inhalers	0.443	0.296	0.665	< 0.001	0.262	0.160	0.430	< 0.001

*Note:* 95% confidence interval (95% CI); minimum wage equivalent to R$1412.00 (MW); chronic obstructive pulmonary disease (COPD).

**FIGURE 1:**
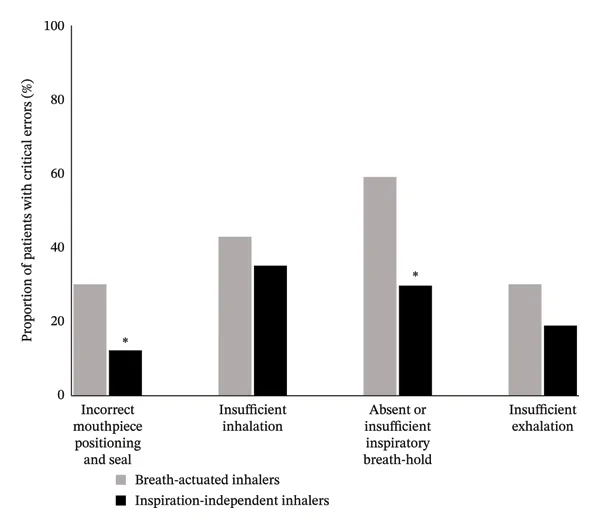
Frequency of critical errors according to inhaler drug delivery mechanism.^∗^ Significant in comparison to the breath‐actuated inhalers group (Pearson’s chi‐square test, *p* < 0.05).

Finally, Figure 1 shows the comparison of the frequency of the main critical inhaler technique errors according to device drug delivery mechanism. The errors of incorrect mouthpiece positioning and seal and absent or insufficient inspiratory breath‐hold were significantly more prevalent among users of breath‐actuated inhalers compared with inspiration‐independent inhalers, with rates of 30.1% vs. 12.2% and 59.1% vs. 29.7%, respectively (*p* < 0.05).

## 4. Discussion

In this cross‐sectional study evaluating the use of inhaler devices in terms of drug administration, we found that only a small proportion of individuals were able to correctly perform all steps of the inhaler technique. The vast majority exhibited one or more critical errors, particularly among older patients and those using a greater number of inhaler devices. Additionally, critical errors were more frequent among users of inspiration‐independent inhalers, with incorrect mouthpiece positioning and insufficient inspiratory breath‐hold being the most prominent. These findings, reflecting real‐world clinical practice and supported by objective assessment methods, highlight the persistent challenges in achieving optimal adherence to inhaled therapy.

Compared with pharmaceutical formulations such as tablets or oral solutions, inhaler devices require a more complex mode of administration, involving multiple steps. Errors in inhaler technique, which are common among patients with asthma or COPD, are associated with poorer clinical outcomes, including exacerbations, hospitalizations, reduced quality of life, and increased healthcare costs [16, 17]. In our study, this challenge was further compounded by the high prevalence of nonadherence to treatment, highlighting the coexistence of two major barriers to effective disease management: inadequate medication adherence and incorrect inhaler technique. Evidence from a recent meta‐analysis has demonstrated that poor adherence to inhaled therapy is significantly associated with an increased risk of COPD exacerbations and worse clinical outcomes [18]. Therefore, the identification and correction of these errors are essential responsibilities of pharmacists and other healthcare professionals [19].

The use of device‐specific checklists is an effective strategy for assessing inhaler technique, enabling the objective detection of errors during practical demonstration [20]. However, the wide variety of inhaler devices available on the market poses challenges to the standardization of assessment, as each device requires distinct procedural steps [21]. To address this limitation, the concept of critical errors has been proposed, defined as failures with a relevant potential to compromise therapeutic effectiveness [12]. Typical examples include inadequate exhalation, poor mouthpiece seal, incorrect inhalation, and absence of a postinhalation breath‐hold [22].

Our findings indicate a high frequency of errors in inhaler technique, with only a small proportion of participants correctly performing all steps and the vast majority committing one or more critical errors. Similarly low rates of correct technique, as low as 6.3%, have been reported in a cross‐sectional study conducted in Pakistan [23], while a study in Iraq [24] reported a nearly identical proportion of 8.6% to that observed in our sample. The prevalence of overall errors and critical errors reported in the literature is consistently high, ranging from 50% to 100% and 14%–92%, respectively [6]. However, a study involving 75 Turkish patients found that 49.3% used their inhaler devices correctly [25]. These discrepancies may be attributed to methodological factors, including heterogeneous definitions of “critical errors” [7, 26], differences in the populations studied [27], and variations in healthcare systems [23]. Disagreements between the two evaluators occurred in 3.5% of assessments and were resolved by a third evaluator.

The results further demonstrate that the occurrence of critical errors is particularly pronounced among older individuals and those using a greater number of inhaler devices. Advanced age has been consistently associated with higher rates of inhaler technique errors [6, 7, 24, 27]. Similarly, the concurrent use of multiple inhaler device models has been identified as a significant risk factor, with patients using two or more devices exhibiting a three‐ to nine‐fold increased likelihood of critical errors [26]. In contrast, Akhoon et al. did not find an association between age and error prevalence, which may be explained by the exclusion of very elderly populations in their study [28], whereas such individuals were included in our sample.

The higher occurrence of critical errors among older individuals and those using multiple inhaler devices may be explained by a combination of pathophysiological, pharmacological, and clinical factors. From a physiological perspective, older patients often exhibit limitations in hand–eye coordination, as well as reduced inspiratory flow and volume, which may hinder the correct use of pMDIs and DPIs, respectively [29]. These limitations are even more pronounced in elderly patients with COPD [7]. With regard to the use of multiple devices, the diversity of operational mechanisms across inhalers may lead to confusion and increased cognitive load, thereby increasing the likelihood of critical errors [6, 26]. In addition, aging is associated with a decline in cognitive functions related to memory, which may impair the acquisition and retention of instructions for correct device use [29]. Many older adults are also exposed to polypharmacy, which may reduce attention to inhaler technique and contribute to inadequate prioritization of respiratory treatment. Furthermore, switching between devices with conflicting instructions may hinder the development of consistent motor memory; for example, DPIs require a rapid inhalation, whereas SMIs require a slow and continuous inhalation.

Additionally, our study identified a higher frequency of critical errors among users of inhaler devices that depend on the patient’s inspiratory effort for drug delivery, particularly DPIs, represented in our study by Aerocaps and Ellipta. These devices require patients to perform a rapid and deep inhalation, followed by an adequate breath‐hold to ensure drug deposition in the lower airways. In our study, critical errors were particularly frequent in steps related to inhaler positioning and postinhalation breath‐hold. These findings are consistent with previous reports demonstrating that errors in inhalation and breath‐hold steps are among the most clinically relevant errors associated with DPI use and may contribute to poorer disease control and an increased risk of exacerbations [3]. In a multicenter study, Kocks et al. reported that incorrect inhaler positioning during inhalation was a recurrent critical error, particularly among Ellipta users, in whom improper positioning compromises the direction of airflow through the lower outlet channels of the device [8]. Similarly, Sanaullah et al. highlighted failure to maintain a postinhalation breath‐hold as one of the most common and clinically significant errors in DPI administration. These findings reinforce that the effectiveness of DPIs is highly dependent on patients’ respiratory capacity and technical ability, making them more vulnerable to errors in populations with cognitive, motor, or functional limitations [23].

In clinical practice, this scenario underscores the need for periodic assessment of inhaler technique, particularly among older patients and those using multiple devices, with treatment tailored to the patient’s functional and cognitive profile. Education on inhaler device use involves multiple device‐specific instructions that vary according to device type, which may represent an additional challenge for older individuals with cognitive and motor limitations. In this context, assessing inhaler technique with a focus on identifying critical errors is particularly useful, as it enables targeted interventions directed at steps whose failure most significantly compromises therapeutic effectiveness. This focused approach facilitates both learning and retention of the essential steps required for proper device use. Training in inhaler technique should not be a one‐time intervention; rather, it requires continuous reassessment at each clinical visit or medication dispensing [30], given that critical errors may develop rapidly with repeated unsupervised use [31]. As many patients obtain their inhaler devices from pharmacies, pharmacists are in a strategic position to identify errors in critical steps, correct them, and reinforce proper technique over time. Evidence indicates that pharmacist‐led educational interventions can substantially reduce the occurrence of errors in inhaler device use [32, 33].

Our study has several limitations. The cross‐sectional design restricts causal inference between inhaler technique errors and clinical outcomes, and the assessment of technique may not necessarily reflect patients’ habitual performance. Additionally, self‐reported information, such as exacerbation history, is subject to recall bias, and the possibility of a Hawthorne effect cannot be excluded. Patients with evident cognitive impairment or requiring assistance with inhaler use were excluded because the study aimed to assess independent inhaler technique. However, as these individuals may be particularly vulnerable to technique errors, their exclusion may have introduced selection bias and potentially underestimated the prevalence of critical errors. The considerable methodological heterogeneity in the definition and categorization of “critical errors,” along with the conduct of the study in only two centers within a single city, may limit the generalizability of the findings. Although the specialized pharmacy serves patients from different levels of care and from both public and private healthcare services across the metropolitan region of Natal, the nonpopulation‐based recruitment strategy may have introduced selection bias, with patients managed exclusively in other healthcare settings potentially underrepresented. Furthermore, potentially relevant clinical and treatment‐related factors, including disease duration, comorbidities, polypharmacy, duration of use of the current inhaler device, and previous device changes, were not assessed, which may have resulted in residual confounding.

On the other hand, the present study is strengthened by its methodological rigor and scientific relevance, as it reflects real‐world clinical practice through the evaluation of a large sample of patients. The objective assessment of inhaler technique, performed through direct observation in a controlled setting, together with the inclusion of multiple device types, minimizes common sources of bias and enhances the applicability of the findings. Future research, preferably longitudinal in design, should further explore the use of critical errors in inhaler technique as a simplified and clinically applicable framework for routine assessment of inhaler use, aiming to facilitate the rapid identification of patients at higher risk of incorrect technique in real‐world settings. In addition, educational interventions aimed at improving correct inhaler use in more vulnerable populations, including pharmacist‐led educational programs, repeated reassessment of technique during routine care or medication dispensing, and strategies to simplify inhalation regimens when multiple devices are prescribed, should be prioritized as key areas for future research.

## 5. Conclusion

This cross‐sectional study, based on the objective evaluation of inhalation technique in a real‐world setting, demonstrated a high prevalence of critical errors among patients with asthma and COPD, particularly in older adults and those using multiple devices. Inhalers dependent on inspiratory capacity, such as Aerocaps and Ellipta, were especially prone to failures, most notably incorrect mouthpiece positioning and an absence of an adequate breath‐hold. These findings underscore the importance of systematic reassessment of inhalation technique and the tailoring of prescriptions according to patients’ functional profiles. Moreover, they highlight the need for continuous educational strategies, with an emphasis on critical steps of inhaler use, particularly among vulnerable populations.

## Funding

This study was supported by the Coordination for the Improvement of Higher Education Personnel (CAPES), Brazil—Funding Code 001.

## Conflicts of Interest

The authors declare no conflicts of interest.

## Supporting Information

Additional supporting information can be found online in the Supporting Information section.

## Supporting information


**Supporting Information** Supporting Materials. Supporting Table. Checklists for assessing inhaler technique. (A) Pressurized metered‐dose inhaler. (B) Dry powder inhaler. (C) Soft mist inhaler.

## Data Availability

The data that support the findings of this study are available from the corresponding author upon reasonable request.
