# Supplementary material for: Critical Errors in Inhaler Technique Among Patients With Asthma and COPD: A Cross‐Sectional Study in a Real‐World Setting
Source: Can Respir J. 2026 Sep 26;2026:8222875. doi: 10.1155/carj/8222875 (PMC13615514; doi:10.1155/carj/8222875)
Supplement: Supplementary file 1 [file CARJ-2026-8222875-s001.docx]

**SUPPLEMENTARY MATERIAL**

Checklists for assessing inhaler technique. (A) Pressurized metered-dose inhaler. (B) Dry powder inhaler. (C) Soft mist inhaler.

(A) INHALER DEVICE HANDLING: PRESSURIZED METERED-DOSE INHALER (pMDI)

| **AEROSOL** | | | |
| --- | --- | --- | --- |
| **STEP** | **ACTIONS** | **YES** | **NO** |
| 1 | Attach the canister to the inhaler and shake the device. |  |  |
| 2 | Remove the inhaler cap. |  |  |
| 3 | Hold the inhaler in a vertical position and place the mouthpiece close to the mouth while keeping an upright posture. |  |  |
| 4 | Exhale deeply. |  |  |
| 5 | Place lips around the mouthpiece ensuring a complete seal. |  |  |
| 6 | Actuate the device at the beginning of a slow and deep inhalation. |  |  |
| 7 | Remove the inhaler while keeping lips closed. |  |  |
| 8 | Perform a post-inhalation breath-hold: 10 seconds or as long as tolerated. |  |  |
| 9 | Exhale away from the inhaler (repeat from step 3 to administer an additional dose if required). |  |  |
| 10 | Close the inhaler. |  |  |
| 11 | Check the dose counter. |  |  |
| 12 | Rinse mouth after inhalation without swallowing the water.* |  |  |
| **This step applies only to inhaler devices containing inhaled corticosteroids (ICS).* | | | |

Source: Adapted from the Diretrizes da Sociedade Brasileira de Pneumologia e Tisiologia para o Manejo

da Asma (2012).

(B) INHALER DEVICE HANDLING: DRY POWDER INHALER (DPI)

| **AEROCAPS®** | | | |
| --- | --- | --- | --- |
| **STEP** | **ACTIONS** | **YES** | **NO** |
| 1 | Remove the inhaler cap. |  |  |
| 2 | Keep the inhaler in an upright position. |  |  |
| 3 | Insert the capsule. |  |  |
| 4 | Close the inhaler. |  |  |
| 5 | Pierce the capsule. |  |  |
| 6 | Keep the head upright. |  |  |
| 7 | Hold the inhaler correctly. |  |  |
| 8 | Exhale normally. |  |  |
| 9 | Place the mouthpiece correctly in the mouth, ensuring lips are sealed and the tongue positioned below. |  |  |
| 10 | Inhale rapidly and deeply through the inhaler. |  |  |
| 11 | Perform an inspiratory breath-hold: 10 seconds or as long as tolerated. |  |  |
| 12 | Exhale away from the inhaler. |  |  |
| 13 | Open the inhaler and check whether the capsule is empty. |  |  |
| 14 | If not empty, repeat the procedure starting from step 4. |  |  |
| 15 | Discard the capsule. |  |  |
| 16 | Close the inhaler. |  |  |
| 17 | Rinse the mouth after inhalation without swallowing the water. |  |  |
| 18 | Clean the device (if necessary). |  |  |

Source: Adapted from the Diretrizes da Sociedade Brasileira de Pneumologia e Tisiologia para o Manejo

da Asma (2012) and the Global Initiative for Asthma (GINA, 2021).

| **ELLIPTA®** | | | |
| --- | --- | --- | --- |
| **STEP** | **ACTIONS** | **YES** | **NO** |
| 1 | Slide the cover fully downward until a click is heard. |  |  |
| 2 | Keep the inhaler in an upright position. |  |  |
| 3 | Confirm dose activation by checking if the dose counter has decreased by one. |  |  |
| 4 | Keep the head upright. |  |  |
| 5 | Hold the inhaler correctly. |  |  |
| 6 | Exhale normally. |  |  |
| 7 | Place the mouthpiece correctly in the mouth, ensuring lips are sealed and the tongue positioned below. |  |  |
| 8 | Inhale slowly and deeply through the inhaler. |  |  |
| 9 | Perform an inspiratory breath-hold: 5 seconds or as long as tolerated. |  |  |
| 10 | Exhale away from the inhaler. |  |  |
| 11 | Clean the device (if necessary). |  |  |
| 12 | Close the device. |  |  |
| 13 | Rinse mouth after inhalation without swallowing the water.* |  |  |
| **This step applies only to inhaler devices containing inhaled corticosteroids (ICS).* | | | |

Source: Adapted from the Diretrizes da Sociedade Brasileira de Pneumologia e Tisiologia para o Manejo

da Asma (2012) and the Global Initiative for Asthma (GINA, 2021).

(C) INHALER DEVICE HANDLING: SOFT MIST INHALER (SMI)

| **RESPIMAT®** | | | |
| --- | --- | --- | --- |
| **STEP** | **ACTIONS** | **YES** | **NO** |
| **Preparation** | | | |
| 1 | Remove the transparent base. |  |  |
| 2 | Insert the cartridge and press firmly until it clicks into place. |  |  |
| 3 | Reattach the transparent base. |  |  |
| 4 | Turn the base in the direction of the arrows on the label. |  |  |
| 5 | Open the cap. |  |  |
| 6 | Point the inhaler toward the ground. |  |  |
| 7 | Press the dose-release button and check for visible mist. |  |  |
| 8 | Close the inhaler. |  |  |
| **Administration** | | | |
| 9 | Turn the base in the direction of the arrows on the label. |  |  |
| 10 | Open the cap. |  |  |
| 11 | Exhale normally. |  |  |
| 12 | Adjust body posture (slightly tilt the head back so the chin points upward). |  |  |
| 13 | Position the mouthpiece between the teeth. |  |  |
| 14 | Place lips around the mouthpiece, ensuring no air leaks. |  |  |
| 15 | Actuate the device. |  |  |
| 16 | Inhale slowly and deeply through the mouth. |  |  |
| 17 | Perform a post-inhalation breath-hold for 10 seconds. |  |  |
| 18 | Wait 15 seconds before repeating actuation, if required. |  |  |

Source: Adapted from the Global Initiative for Chronic Obstructive Pulmonary Disease (GOLD, 2021) and Newman (2005).
